# Deciphering the Molecular Mechanism Responsible for Efficiently Inhibiting Metastasis of Human Non-Small Cell Lung and Colorectal Cancer Cells Targeting the Matrix Metalloproteinases by *Selaginella repanda*

**DOI:** 10.3390/plants10050979

**Published:** 2021-05-14

**Authors:** Mohd Adnan, Arif Jamal Siddiqui, Walid Sabri Hamadou, Mejdi Snoussi, Riadh Badraoui, Syed Amir Ashraf, Arshad Jamal, Amir Mahgoub Awadelkareem, Manojkumar Sachidanandan, Sibte Hadi, Mushtaq Ahmad Khan, Mitesh Patel

**Affiliations:** 1 Department of Biology, College of Science, University of Hail, Hail P.O. Box 2440, Saudi Arabia; drmohdadnan@gmail.com (M.A.); arifjamal13@gmail.com (A.J.S.); walidsabrimail@gmail.com (W.S.H.); snmejdi@yahoo.fr (M.S.); badraouir@yahoo.fr (R.B.); arshadjamalus@yahoo.com (A.J.); 2 Section of Histology-Cytology, Medicine Faculty of Tunis, University of Tunis El Manar, La Rabta-Tunis 1007, Tunisia; 3 Department of Clinical Nutrition, College of Applied Medial Sciences, University of Hail, Hail P.O. Box 2440, Saudi Arabia; amirashrafy2007@gmail.com (S.A.A.); mahgoubamir22@gmail.com (A.M.A.); 4 Department of Oral Radiology, College of Dentistry, University of Hail, Hail P.O. Box 2440, Saudi Arabia; smanojk68@gmail.com; 5 School of Forensic and Applied Sciences, University of Central Lancashire, Preston PR1 2HE, UK; shadi@uclan.ac.uk; 6 Department of Microbiology and Immunology, College of Medicine and Health Sciences, UAE University, Al Ain 17666, United Arab Emirates; 7 Bapalal Vaidya Botanical Research Centre, Department of Biosciences, Veer Narmad South Gujarat University, Surat 394230, India

**Keywords:** *Selaginella repanda*, non-small cell lung cancer, colorectal cancer, matrix metalloproteins, tissue inhibitor matrix metalloproteinase, metastasis, gene expression

## Abstract

*Selaginella* species are known to have antimicrobial, antioxidant, anti-inflammatory, anti-diabetic as well as anticancer effects. However, no study has examined the cytotoxic and anti-metastatic efficacy of *Selaginella repanda (S. repanda)* to date. Therefore, this study aimed to evaluate the potential anti-metastatic properties of ethanol crude extract of *S. repanda* in human non-small-cell lung (A-549) and colorectal cancer (HCT-116) cells with possible mechanisms. Effect of *S. repanda* crude extract on the growth, adhesion, migration and invasion of the A-549 and HCT-116 were investigated. We demonstrated that *S. repanda* crude extract inhibited cell growth of metastatic cells in a dose and time dependent manner. Incubation of A-549 and HCT-116 cells with 100–500 µg/mL of *S. repanda* crude extract significantly inhibited cell adhesion to gelatin coated surface. In the migration and invasion assay, *S. repanda* crude extract also significantly inhibited cellular migration and invasion in both A-549 and HCT-116 cells. Moreover, reverse transcription-polymerase chain reaction, and real-time PCR (RT-PCR) analysis revealed that the activity and mRNA level of matrix metalloproteinase-9 (MMP-9), matrix metalloproteinase-2 (MMP-2) and membrane type 1-matrix metalloproteinase (MT1-MMP) were inhibited. While the activity of tissue inhibitor matrix metalloproteinase 1 (TIMP-1); an inhibitor of MMPs was stimulated by *S. repanda* crude extract in a concentration-dependent manner. Therefore, the present study not only indicated the inhibition of motility and invasion of malignant cells by *S. repanda*, but also revealed that such effects were likely associated with the decrease in MMP-2/-9 expression of both A-549 and HCT-116 cells. This further suggests that *S. repanda* could be used as a potential source of anti-metastasis agent in pharmaceutical development for cancer therapy.

## 1. Introduction

Over the past few years, cancer is the leading cause of death globally, resulting in about 9.6 million deaths in 2018. In men, commonly occurring cancers are lung, prostate, colorectal, stomach and liver, whereas, in women, cervical, lung, colorectal and thyroid cancers are most common [1]. Metastasis is the most devastating hallmark of cancer with more than 90% of deaths worldwide and a major obstacle in the treatment of cancers, which is defined as the emergence of secondary and tertiary tumors in tissues and organs aside from the origin [2,3]. Options available for the treatment of various types of cancers are chemotherapy, radiation therapy and surgery. All of these treatment options have harmful and severe adverse effects [4,5]. Therefore, in recent times, back to nature approach is highly accepted. A large number of plant-based molecules are developed and approved for anticancer and anti-metastatic therapies [6,7]. Hence, to find out the feasible course of action to have safe and to lower the adverse effects prompted by chemotherapy, the continuous exploration for anticancer phytochemicals plays a critical role.

The cosmopolitan genus *Selaginella* P. Beauv. also acknowledged as a “spike moss” belongs to the family *Selaginellaceae*, possessing about 700 to 750 species distributed around the globe. The *Selaginella* plants are usually used by a tribal community to cure sore throat, hepatic disorders, fever, cirrhosis, jaundice, cholecystitis, cough of lungs, diarrhea and various other related ailments. Moreover, it is also used for promoting the blood circulation, removing blood stasis, prevention of external bleeding after separation of the umbilical cord and trauma [8,9]. This is due to the presence of high content of different phytochemicals including, carbohydrates, chromones, benzenoids, alkaloids, coumarins, lignans, flavonoids, phenylpropanoids, steroids, pigments and quinoids [10,11,12,13,14,15,16,17,18,19]. Although the members of *Selaginella* are reported for their efficient medicinal applications, the cytotoxicity and anti-metastatic activity of *S. repanda* (Desv. &Poir.) against lung and colon cancers have not been described. Furthermore, matrix metalloproteinases (MMPs) are believed to be intricated in cancer development and progression. MMPs are known to play a vital role in tumor metastasis, angiogenesis and invasion, and are considered as a potential anticancer target. Therefore, the present study aimed to find out the cytotoxic and anti-metastatic efficacy of traditionally treasured plant *S. repanda* on human non-small cell lung and colorectal cancer with possible mechanisms explaining the expression of MMP-2/-9, MT1-MMP and TIMP-1 with and without treatment with *S. repanda.*

## 2. Results

### 2.1. High Resolution-Liquid Chromatography-Mass Spectroscopy (HR-LC-MS) Identification of S. repanda Phytoconstituents

Comprehensive phytoconstituent analysis from crude extract of *S. repanda* was carried out to analyze the plant’s total extract obtained from 85% aqueous ethanol obtained from the whole plant of *S. repanda* via UHPLC-PDA-ESI-MS/MS. A diverse class of bioactive compounds was revealed, which are known to be potent and linked to various biological activities. Table 1 listed the identified phytoconstituents in *S. repanda* crude extract and are in agreement with previous studies [20,21]. A total of fifty-four constituents were tentatively identified. The molecular mass, mass spectral fragmentation patterns of the constituents, and standard samples were used for the purpose. Out of all the identified compounds, phenols and flavonoids were the major constituents. Identified phenols were isoferulic acid, chlorogenic acid, 4-coumaric acid, 7-hydroxycoumarine, coumarin, caffeic acid, formononetin, 4-methoxycinnamic acid and 2-amino-1,3,4-octadecanetriol. Identified potent flavonoid compounds were glycitin, vitexin, rhamnetin, kaempferol, luteolin, rutin, diosmetin, apigenin H, genistein. Identified potent terpenoids, alkaloids, benzenoids, monocarboxylic acid and ester compounds were urocanic acid, guvacoline, hordenine, sedanolide, scopoletin, norharman, citral, methylcinnamate, 8-hydroxyquinoline, pulegone and 4-hydroxyphenylacetic acid [9]. 

Overall, LC-MS analysis endorsed the rich nature of the *S. repanda* plant in terms of varying chemical classes of compounds present in it, whereby the majority of all the identified compounds are flavonoids and phenolics in nature as previously described [9,20,21]. Flavonoids and natural phenolic acids are one of the most prevalent and pharmacologically active groups of plant secondary metabolites. Whereas, alkaloids are nitrogenous compounds that are widely distributed from prokaryotes to eukaryotes and are well-known for their different biological activities like anti-microbial, anti-HIV, anticancer and antiparasitic. These compounds play a crucial role in the prevention of cancer via various mechanisms at a molecular level. This may include impeding the signaling pathways, migration, differentiation and proliferation inhibition, gene regulation, carcinogen metabolism, induction of apoptosis via arresting cell cycle, etc. [22,23]. Therefore, the presence of diverse biologically active constituents contributed immensely to the plant’s bioactive potential, and perhaps the ensuing better anticancer and anti-metastatic bioactivities. 

### 2.2. Cytotoxic Effect of S. repanda Crude Extract

**Figure 1:**
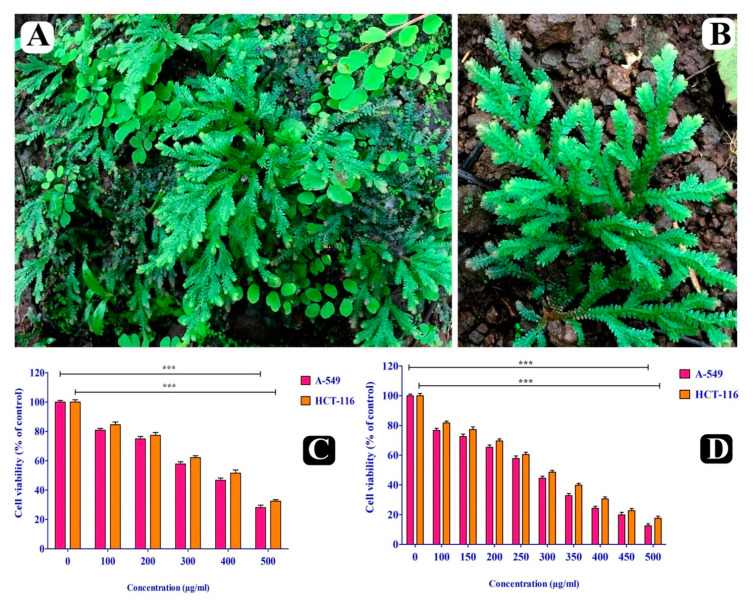
*S. repanda* plant and its anticancer activity. (**A**) Plant in wild (**B**) Close view of plant (**C**) Anticancer activity of *S. repanda* crude extract against A-549 and HCT-116 cancer cells for 24 h. (**D**) Anticancer activity of *S. repanda* crude extract against A-549 and HCT-116 cancer cells for 48 h. Error bars indicate SDs (± standard deviation) of three independent experiments. Significance; ns > 0.05, * *p* < 0.05, ** *p* < 0.005, *** *p* < 0.0005.

Cytotoxicity of *S. repanda* crude extract was evaluated against A549 and HCT-116 cancer cell lines by MTT assay at 24 and 48 h. Significant inhibition of both cancer cells’ viability was observed in a time and dose-dependent manner. The IC_50_ values for 24 h were found to be 341.1 μg/mL and 378.8 μg/mL (Figure 1C) and for 48 h were found to be 326.7 μg/mL and 351.4 μg/mL for A549 and HCT-116 cells (Figure 1D).

### 2.3. Anti-Migratory Effect of S. repanda Crude Extract

**Figure 2:**
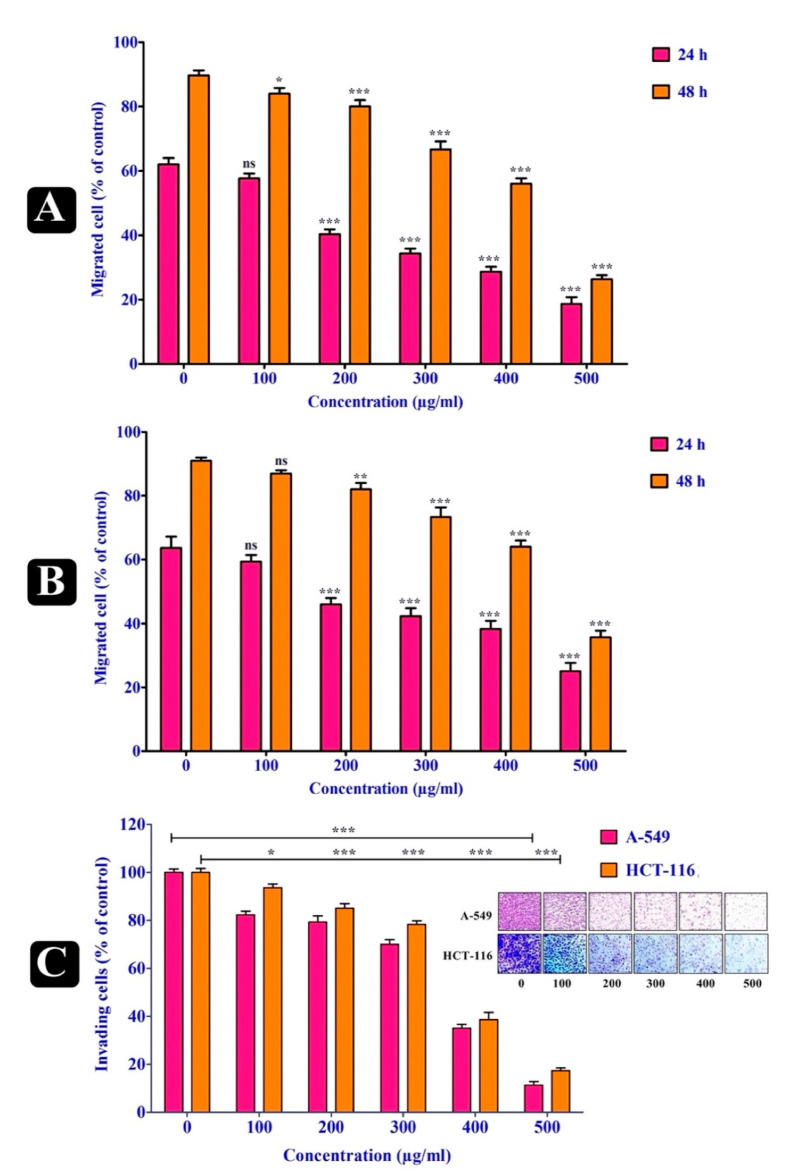
Anti-migration and anti-invasion effects of *S. repanda* crude extract. (**A**) Anti-migration activity on A-549 cancer cells (**B**) Anti-migration activity on HCT-116 cancer cells. The number of migrated cells was quantified in five different fields from three independent experiments. (**C**) Anti- invasion activity on A-549 and HCT-116 cancer cells. The invading cells were counted in five random fields under microscopes. Error bars indicate SDs (± standard deviation) of three independent experiments. Significance; ns > 0.05, * *p* < 0.05, ** *p* < 0.005, *** *p* < 0.0005.

The most important metastatic event is the motility of cells, which takes place in different epithelial cells during the progression of cancer. Wound closure assay was carried out to study the effect of *S. repanda* crude extract on the migration of cells over wound scratch, made on culture plates. The migration of both A549 and HCT-116 cancer cells was evidently inhibited by the treatment of *S. repanda* crude extract in a time as well as dose-dependent manner, when compared to untreated control cells. Hence, *S. repanda* crude extract evidently inhibited the anchorage and spread of both cell lines along the edge of wound scratch (Figure 2A,B).

### 2.4. Anti-Invasion Effect of S. repanda Crude Extract

Transwell^®^ cell culture inserts coated with Matrigel matrix were used to evaluate the ability of *S. repanda* crude extract to inhibit the invasion of A549 and HCT-116 cells, following treatment with different concentrations (100–500 μg/mL). The cellular invasion of both A549 and HCT-116 cancer cells was significantly inhibited by the treatment of *S. repanda* crude extract (100 μg/mL) in a time as well as dose-dependent manner, when compared to untreated control cells (Figure 2 C).

### 2.5. Anti-Adhesion Effect of S. repanda Crude Extract

**Figure 3:**
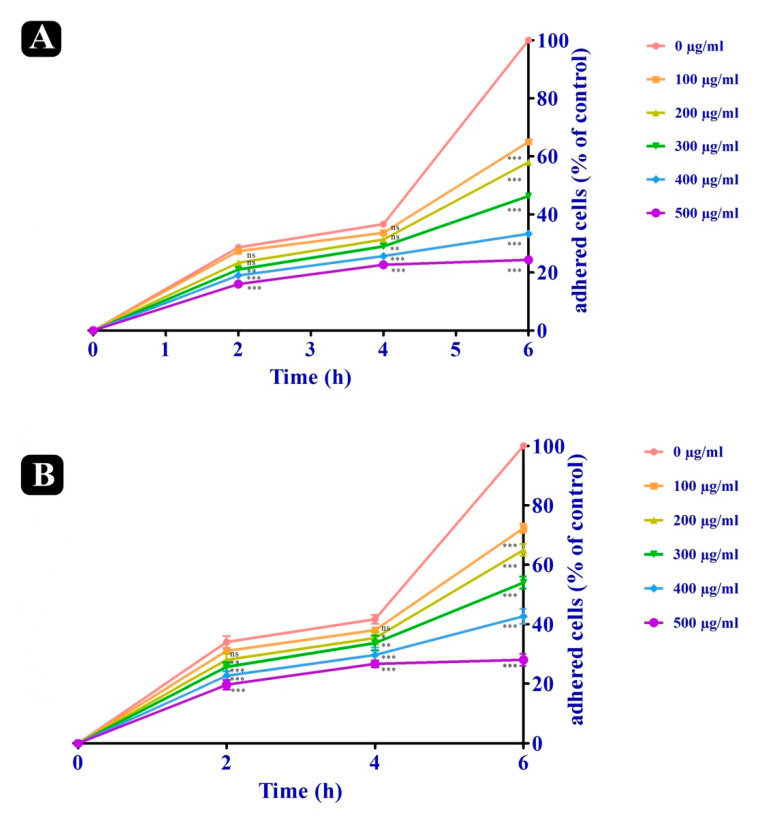
Anti-adhesion effects of *S. repanda* crude extract on gelatin coated surfaces. (**A**) Anti-adhesion activity on A-549 cancer cells. (**B**) Anti-adhesion activity on HCT-116 cancer cells. Error bars indicate SDs (± standard deviation) of three independent experiments. Significance; ns > 0.05, * *p* < 0.05, ** *p* < 0.005, *** *p* < 0.0005.

Adhesion to endothelial cells by malignant cells is known to be mediated by extracellular matrix proteins (ECM), thereby, we evaluated the adhesion property of both A549 and HCT-116 cancer cells to gelatin coated surfaces both in the presence and absence of *S. repanda* crude extract. The adhesion of A549 and HCT-116 cells were reduced by 75.67% and 72.0% (500 μg/mL) at the incubation of 6 h. Relative adherence was measured by setting the number of adherent cells at 6 h to 100% (Figure 3A,B). 

### 2.6. Changes in Transcriptional Level of Metastasis Related Genes 

**Figure 4:**
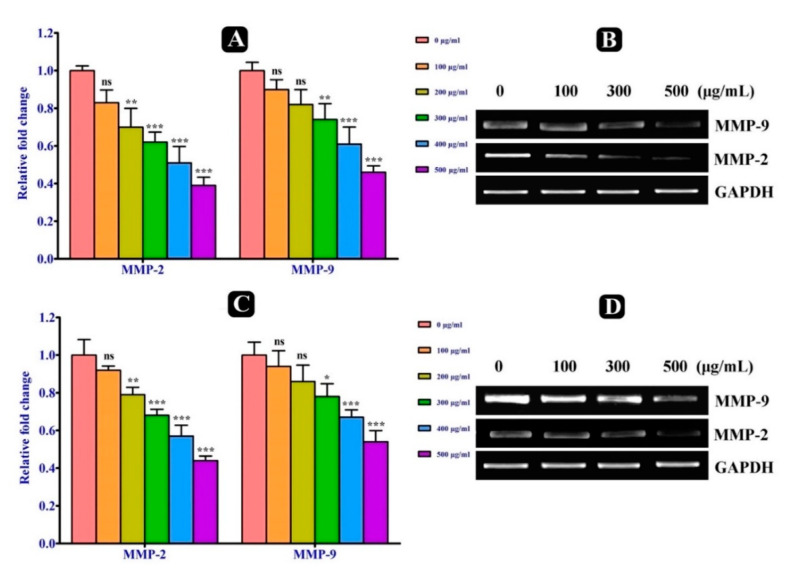
Effect of *S. repanda* crude extract on metastasis related genes. (**A**) Reverse transcription PCR to analyze the mRNA expression level of MMP-2 and MMP-9 in A-549 cancer cells. (**B**) Quantitative real-time PCR. (**C**) Reverse transcription PCR to analyze the mRNA expression level of MMP-2 and MMP-9 in HCT-116 cancer cells. (**D**) Quantitative real-time PCR. Significance; ns > 0.05, * *p* < 0.05, ** *p* < 0.005, *** *p* < 0.0005.

**Figure 5:**
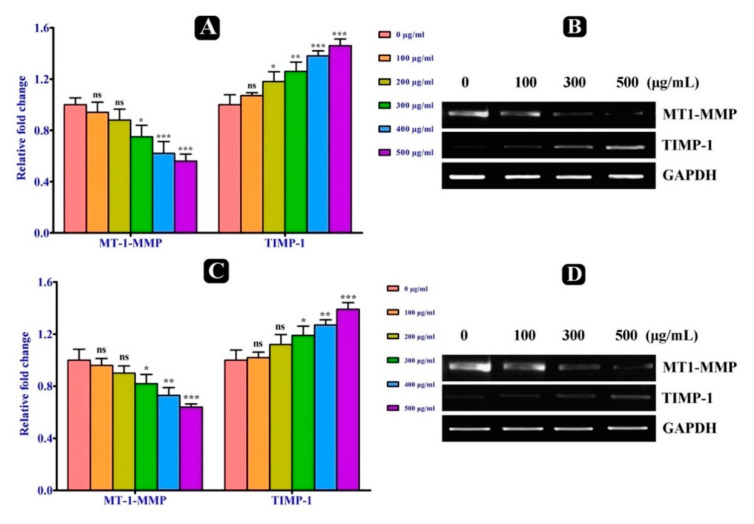
Effect of *S. repanda* crude extract on metastasis related genes. (**A**) Reverse transcription PCR to analyze the mRNA expression level of MT1-MMP and TIMP-1 in A-549 cancer cells. (**B**) Quantitative real-time PCR. (**C**) Reverse transcription PCR to analyze the mRNA expression level of MT1-MMP and TIMP-1 in HCT-116 cancer cells. (**D**) Quantitative real-time PCR. Significance; ns > 0.05, * *p* < 0.05, ** *p* < 0.005, *** *p* < 0.0005.

The expression level of metastasis related genes MMP-2, MMP-9, MT1-MMP and TIMP-1 in both A549 and HCT-116 malignant cells, which were induced by *S. repanda* crude extract were determined by real time PCR. Firstly, the expression level of MMP-2 and MMP-9 genes were evaluated and was found to be decreased in both cells treated with *S. repanda* crude extract after 24 h incubation, when compared to untreated cells (Figure 4A–D). After the analysis of MMP-2 and MMP-9, we further examined the effect of *S. repanda* crude extract on the expression of activator and inhibitor genes of MMPs (MT1-MMP and TIMP-1). The expression level of TIMP-1 was found to be increased and of MT1-MMP gene was decreased in both cells after the treatment of *S. repanda* crude extract, when compared to untreated cells after 24 h incubation (Figure 5A,B). These results show that *S. repanda* crude extract can regulate the expression of MMP-2 and MMP-9 genes, which can possibly further control the cascade of metastasis.

## 3. Discussion

Regardless of all current progress in oncology, cancer is still one of the most life-threatening diseases around the globe [1]. It takes place as a localized disease, but can extend to different sites of the human body through migration, invasion and metastasis [2]. Metastasis is a multifaceted process involving an array of complicated mechanisms, which begins with extrication, accumulation and motility of cancer cells, followed by sticking to endothelial cells and the start of cancerous growth at different sites [24]. Progression of metastasis occurs after the degradation of ECM with cancer cells via different proteases like serine proteinase, cathepsins, MMPs and plasminogen activator, which prompts the separation of the intercellular matrix to promote the mobility of cancer cells [25]. Amongst involved proteases, MMP-9 and MMP-2 are profoundly implicated in cancer invasion and metastasis as they are the most essential for the degradation of base membranes [26,27,28].

Due to resistance to apoptosis and cytotoxic agents, metastasis is the dominant basis for cancer associated death. The percentage of morbidity and mortality in metastatic cancer patients is high, due to the failure of current chemotherapy agents to selectively and effectively kill cancer cells without destroying healthy cells at the sites of metastasis [29]. Metastasis is still a crucial clinical challenge in cancer treatment for researchers around the globe. Up till now, no such strong cleanse for cancer and its other catastrophic presentations have been found [29]. Currently, radiation therapy, chemotherapy, surgery are the conventional treatments for cancer. These treatments come with a range of side effects to human health; therefore, the importance of traditional medicines may decline. Thus, metastasis found the greatest challenging obstacle for successful cancer management and can be viewed as the last edge of cancer research [24].

Since ancient times, natural products have been recognized as an excellent source of bioactive compounds. They have been the primary source of general medicines, and can also be used directly as medicines [30,31,32,33,34,35]. Therefore, the pursuit and curiosity in the identification of medicinal plants and their derived natural products for evolving the novel cancer therapeutic strategies expanded vastly in recent times. In our previous study [9], a comprehensive phytochemical analysis from the crude extract of *S. repanda* was carried out via HR-LC-MS. A chromatogram was obtained with both positive and negative run and different types of phytochemicals were identified. In-depth profiling revealed various classes of metabolites such as alkaloids, flavonoids, sugars, vitamins, amino acids, phenols, terpenoids, phenols, etc. Though their biological activities are known, it can be evidently said that these compounds are somehow directly or indirectly responsible for the possible anti-metastatic effect on lung and colon cancer cells. However, individual testing of each identified phytochemical is required to reveal and interpret the actual involvement of these *S. repanda* compounds in anti-metastasis.

Plants in the genus *Selaginella* have diverse therapeutic potential on different cancer cells, comprising induction of apoptosis, inhibition of cell proliferation and arrest of cell cycle. There are species of *Selaginella* (*S. delicatula*, *S. tamariscina*, *S. moellendorffii*), which are known to possess potent antioxidant and antitumor activities, related to activation of apoptosis through DNA fragmentation and nucleus clotting [36], by inducing the expression of p53 and G1 arrest [37], through obstruction in fatty acid synthesis [38], inhibiting transactivation of iNOS and COX-2 via inactivating the NF-kB and avoiding the p65 translocation [39]. Several studies revealed the potent antitumor activities by *S. uncinata* and *S. tamariscina*, while moderate activity was found against Bel-7402 and HeLa cells by *S. moellendorfii*. Furthermore, out of all *Selaginella* species in the world, *S. tamariscina* is considered one of the most biologically potent plants, which is very well-known to inhibit the growth of various cancers (breast, leukemia, gastric, lung) [36,37,38,39]. Additionally, in osteosarcoma cells, it also possesses the anti-metastatic activity by down-regulating the MMP-2 and MMP-9 secretions and increasing the TIMP-1 and TIMP-2 expressions via Akt-dependent and p38 pathways [37,38,40,41,42]. 

A similar study with *S. delicatula* revealed the cytotoxicity against cancer cells of P-388, HT-29 [43] Raji, Calu-1, lymphoma and leukemia [44], due to the presence of phytocompounds robustaflavone and amentoflavone or its derivatives. Few other studies using *S. moellendorfii* show the growth inhibition of OVCAR-3, HeLa and FS-5 cancer cells [45,46], and anti-metastasis activity in lung cancer cells [39]. However, no studies on cytotoxicity and anti-metastasis of *S. repanda* crude extract against human cancer cells exist to date, and the mechanism of anticancer potential also remains unclear. Therefore, our study was designed to potentially identify the anti-metastatic activity of *S. repanda* crude extract against A549 and HCT-116 cancer cells with a possible molecular mechanism. 

*S. repanda* crude extract displayed significant cytotoxic potential on both malignant A549 and HCT-116 cancer cells, and their IC_50_ were 341.1 μg/mL and 378.8 μg/mL, respectively. We performed an in vitro wound healing assay to evaluate the effect of *S. repanda* crude extract on cell migration, as migration is an important event in the progression of metastasis and cancer. *S. repanda* crude extract significantly inhibited the migration of cancer cells in the direction of the wounded area. Such results of the present study displayed that *S. repanda* crude extract impeded cell migration, which is crucial during the early phase of wound healing. Furthermore, *S. repanda* crude extract also remarkably retarded the invasion of both malignant cancer cells. Therefore, in the present study using wound healing, adhesion and invasion assays, we have shown that *S. repanda* crude extract efficiently inhibits the metastasis in both malignant cells in vitro.

To further elucidate the possible molecular mechanism behind the anti-metastatic potential of *S. repanda* crude extract, the mRNA expression level of MMP-2 and MMP-9 genes involved in the metastasis process was investigated. Both MMP-2 and MMP-9 genes mRNA expression levels were significantly decreased in a dose-dependent manner in both malignant cells. In metastasis, MMP-9 is considered the most significant protease, and its expression is connected with the growth of local tumor, invasion and metastasis in the majority of the carcinomas [47]. Therefore, increasing evidence suggests that a particular suppression of MMP-9 activity might avert metastasis [48]. Moreover, the expression of MMP genes is mainly controlled by their activators and inhibitors at the transcriptional, post-transcriptional and at the protein level [26,49]. TIMPs are assumed to play an immense role in the inhibition of MMPs [50]. In the present study, A549 and HCT-116 cells treated with *S. repanda* crude extract collectively up-regulated the expression of TIMP-1 gene. Apart from TIMP-1, MT1-MMP is another key enzyme among the regulation of MMPs, whose overexpression has the main effect on the growth of tumors [51]. It is mainly responsible for the activation of MMP2 [52,53]. The expression of MT1-MMP gene was significantly found to be inhibited in both A549 and HCT-116 cells treated with *S. repanda* crude extract. Hence, these results revealed that anti-metastatic effect of *S. repanda* crude extract is linked to the inhibition of enzymatically degradative processes of tumor metastasis.

Natural products are produced by all organisms, but plants are the major contributors. All of these organisms co-exist in the ecosystem and interact with each other in various ways in which chemistry plays a major role. Various approaches to understand the taxonomy of plants have been evolved over the years, which include morphological, anatomical and chemotaxonomic classification. However, morphological and anatomical classification system is considered as traditional approach, whereas, the science of chemotaxonomy or chemical taxonomy is a modern approach to classify the plants, especially on the basis of their chemical constituents [54,55]. The phenolics, alkaloids, terpenoids and non-protein amino acids are the four important and widely exploited groups of compounds utilized for chemotaxonomic classification [56]. These groups of compounds exhibit a wide variation in chemical diversity, distribution and function [56,57]. From this study, *S. repanda* have been recognized as an excellent source of phytoconstituents with diverse chemical class, hence our data can also be used by ethnopharmacologists, taxonomists and ethnobotanists for chemotaxonomic importance to solve the selected taxonomical problems.

Furthermore, as we stated earlier that our study is the first study to report the phytochemistry of *S. repanda* and the identified constituents can be seen in Table 1. In the literature, around 130 chemically defined natural products are reported from 32 species of *Selaginella*, which are belonging to the classes of pigments, benzenoids, alkaloids, carbohydrates, coumarins, flavonoids, chromones, oxygen heterocycle, lignans, phenylpropanoids, quinoids and steroids. Here, we have discussed the phytoconstituents of other *Selaginella* species in comparison to *S. repanda*. Chao et al. 1987 identified hordenine, hordenine-[6-O-(4-hydroxy-cinnamoyl)-β-D-glucosyl]-(1,3)-α-L-rhamnoside, hordenine-O-α-L-rhamnopyranoside, -methyltyramine-O-α-L-rhamnoside as alkaloids in *S. doederleinii* [58]. Similarly, in *S. moellendorfii*, identified alkaloids were selaginellic acid, 5-hydroxyselaginellic acid, 5-hydroxy-N8,N8-dimethylpseudophrynaminol, *N*-selaginelloyl-*L*-phenylalanine, *N*-(5-hydroxyselaginelloyl)-*L*-phenylalanine, neoselaginellic acid, and *N*-(5-Hydroxyneoselaginelloyl)-*L*-phenylalanine [59]. In *S. tamariscina,* adenosine and guanosine were identified as alkaloids [60]. Likewise, 4-hydroxy-benzoic acid [61], arbutin, vanillic acid, syringic acid [60,62] are identified as benzoids in *S. pulvinata* and *S. tamariscina* respectively. 

Additionally, in carbohydrate class, selaginose was identified in *S. adunca*, *S. asperula*, *S. epirrhizos*, *S. galeotti*, *S. geniculata*, *S. kraussiana*, *S. marginata*, *S. parkeri*, *S. plumosa*, *S. sanguinolenta*, *S. stellate* and *S. sulcata* [63]; 2-carboxy-arabinitol was identified in *S. mertensii* [64]; and mycose was identified in *S. pulvinata* [61]. Moreover, 8-methyl-eugenitol, uncinoside A, uncinoside B was identified as chromone in *S. uncinata* [65,66]. On the other hand, numerous flavonoids were identified in various Selaginella species. For example, 2,3-dihydroamentoflavone, 2″,3″-dihydroamentoflavone, tetrahydro-amentoflavone was found in *S. bryopteris* [67]; amentoflavone-7,4,7,4-tetramethylether was found in *S. moellendorfii* [68]; 4′,7″-di-O-methyl-amentoflavone was found in *S. sinensis* [69]; 4′,7″-di-O-methyl-amentoflavone was found in *S. willdenowii* [70]; amentoflavone was identified in various species of Selaginella i.e., *S. braunii*, *S. davidii*, *S. delicatula*, *S. denticulata*, *S. kraussiana*, *S. moellendorfii*, *S. pulvinata*, *S. rupestris*, *S. sanguinolenta*, *S. selaginoides*, *S. sinensis*, *S. stauntoniana*, *S. tamariscina*, *S. uncinata*, *S. willdenowii* [44,69,70,71,72,73,74,75]; apigenin-7-O-β-neohesperidoside, apigenin-8-C- β-D-glucopyranoside, 6,8-di-C-β-D-glucopyranosyl-apigenin, 6-C-β-D-glucopyranosyl-8-C-β-D-xylopyranosyl-apigenin, 6-C-β-D-xylopyranosyl-8-C-β-D-glucopyranosyl-apigenin was identified in *S. moellendorfii* [76,77]; 2″,3″-dihydro-4′,7,7″-trimethylether-robustaflavone, 2,3-dihydro-4′,7,7″-trimethylether-robustaflavone, 2″,3″-dihydro-4′,7,-dimethylether-robustaflavone, 4′,7-dimethylether-robustaflavone, 4′-methylether-robustaflavone was identified in *S. delicatula* [44,78]; and sumaflavone was identified in *S. tamariscina* [79]. 

Lignan was also found in other *Selaginella* species. 5-acethyl-dihydro-2-(3′,5′-dimethoxy-4′-hydroxy-phenyl)-7-methoxybenzofuran was found in *S. tamariscina* [60]; (-)-lirioresinol A, (-)-lirioresinol B, (+)-matairesinol was found in *S. doederleinii* [18]; syringaresinol, tamariscinoside B, tamariscinoside C was found in *S. tamariscina* [60,62]. Some important pigments are also known to be found in various *Selaginella* species, i.e., selaginellin in *S. sinensis* [80], selaginellin A, selaginellin B in *S. tamariscina* [81], selaginellin C, selaginellin D, selaginellin E, selaginellin F, selaginellin G, selaginellin H in *S. pulvinata* [82,83,84]. Quinoids are also known to be found in some *Selaginella* species. Chrysophanic acid, emodin, physcion are identified in *S. stauntoniana* [85]; 1-methoxy-3-methylanthraquinone was identified in *S. tamariscina* [85]. Similarly, steroids are also known to be present in numerous *Selaginella* species, i.e., cholesterol, 22-dehydrocampesterol in *S. delicatula*, *S. doederleinii* [86], 3β-16α-dihydroxy-(5α)-cholestan-21-oic acid in *S. pulvinata* [87], 24α-ethyl-cholest-5-en-3β-ol, 24α-methyl-cholest-5-en-3β-ol, 24β-methyl-cholest-5-en-3β-ol, 24α-ethyl-cholesta-5,22-dien-3β-ol in *S. delicatula* and *S. doederleinii* [86] and β-sitostero in *S. doederleinii*, *S. moellendorfii* and *S. pulvinata* [61,88,89]. Therefore, our results can also be correlated and compared with the possibility to identify other *Selaginella* species for their anti-metastatic and anticancer efficacy against various carcinomas, due to the presence of diverse and potential phytoconstituents.

## 4. Materials and Methods

### 4.1. Plant Material Collection and Extraction 

Whole plant of *S. repanda* was collected from the wild regions of Gujarat state, India during the July–August period of 2019. The voucher specimen (BVBRC035) was deposited at Bapalal Vaidya Botanical Garden, Department of Biosciences, Veer Narmad South Gujarat University, Surat, Gujarat, India. The collected plant material was dried in an oven and then grounded into fine powder followed by storage in airtight containers. A total of 20 g of *S. repanda* powder was soaked in 85% ethanol for 24 h at 37 °C with vigorous shaking. The ethanol phase was filtered with Whatman no. 1 filter paper and then concentrated using a rotary evaporator to get the dried residue. All the assays were performed using a stock solution of crude ethanol extract.

### 4.2. HR-LC–MS Analysis

Phytochemistry of *S. repanda* crude extract was analyzed using UHPLC-PDA-Detector Mass Spectrophotometer (HR-L = CMS 1290 Infinity UHPLC System), Agilent Technologies^®^, Santa Clara, CA, USA. The liquid chromatographic system consisted of the HiP sampler, binary gradient solvent pump, column compartment and quadrupole time of flight mass spectrometer (MS Q-TOF) with the dual Agilent Jet Stream Electrospray (AJS ES) ion source. Of the sample, 10 µL was injected into the system, followed by separation in the SB-C18 column (2.1 mm × 50 mm, 1.8 µm particle size). Solvent A (1% formic acid in deionized water) and solvent B (acetonitrile) were used as solvents. A flow rate of 0.350 mL/min was used, while MS detection was performed in MS Q-TOF. Compounds were identified via their mass spectra and their unique mass fragmentation patterns. Compound Discoverer 2.1, ChemSpider and PubChem were used as the main tools for the identification of the phytochemical constituents [9].

### 4.3. Cell culture and Treatment

Human lung (A549) and colon (HCT-116) cancer cell lines were obtained from National Centre for Cell Science (NCCS), India and propagated in a humidified atmosphere with 5% CO_2_ at 37 °C. Cells were maintained in 25 cm^2^ flask having Dulbecco’s Modified Eagle’s Medium (DMEM) supplemented with 10% Fetal Bovine Serum (FBS). They were grown up to 80% confluence for future analyses. Cells were treated with different concentrations of *S. repanda* crude extract (100–500 μg/mL).

### 4.4. Cell Viability Analysis Using MTT Assay

To determine the cytotoxicity of *S. repanda* crude extract, an MTT colorimetric assay was performed. For seeding, 96-well plates were used for both human cancer cell lines with incubation in humidified atmosphere comprising of 5% CO_2_ at 37 °C up to adherence. Different concentrations of *S. repanda* crude extract (100–500 μg/mL) were then used to treat the cells for 24 h, followed by washing with PBS solution. Cells were then subjected with 100 μL of MTT solution (3-(4,5-dimethylthiazolyl-2)-2,5-diphenyltetrazolium bromide) (5 mg/mL), followed by 4-h incubation. The medium was then removed and to solubilize the formazan crystals, 100 µL of dimethyl sulfoxide (DMSO) was added. Using ELISA reader, the amount of formazan crystal was determined by measuring the absorbance at 570 nm. All assays were carried out in triplicate, and 50% cytotoxic concentration (IC_50_) was calculated. 5-Fluorouracil was used as a positive control.

### 4.5. Wound Closure Assay 

The effect of *S. repanda* crude extract on the motility of A-549 and HCT-116 cells was carried out using the wound healing assay. Confluent monolayer cell culture grown in 6 well plates was used to perform the assay. Cells were seeded at 1 × 10^6^ cells/mL in 3 mL final volume of growth medium. With the help of a sterile 1 mL pipette tip, an injury line was made in the central area of culture. After washing with phosphate-buffered saline (PBS), DMEM medium containing 2% FBS with and without *S. repanda* crude extract (100–500 μg/mL) was added into the well, floating cells were discarded. Plates were then incubated for 48 h at 37 °C. Wound closure through cell migration was measured at 0, 24 and 48 h under a microscope. The cells migration towards the wound scratches was expressed as migrated cells percentage and calculated as [90]:

Migrated cells percentage = [(At = 0 h − At = Δh)/At = 0 h] × 100(1)

where, 

At = 0 h is the area of wound measured immediately after scratching

At = Δh is the area of wound measured 24 or 48 h after scratching

### 4.6. Invasion Assay

The effect of *S. repanda* crude extract on the invasion ability of A549 and HCT-116 cells were observed using Transwell^®^ chambers with 6.5 mm polycarbonate filters of 8 μm pore size (TCP152, Himedia^®^, India). A 50 μL aliquot of the Matrigel (E1270, Merck^®^, India) was pipetted into the upper chamber (culture insert), which was placed in a lower chamber with sterile forceps and incubated for the solidification of the gel at 37 °C for 30 min. Cells were seeded into the upper chamber with 1 × 10^6^ cells/mL consisting of 100 μL of serum free media. Immediately this chamber was then transferred into the lower chamber consisting of 600 μL of medium (2% FBS) with and without *S. repanda* crude extract (100–500 μg/mL). The plates were then incubated for 24 h at 37 °C. Afterward, the upper chamber was taken out, and using the cotton buds, non-migrated cells on its inner surface were wiped off. Furthermore, the migrated cells on the outer surface of the upper chamber were fixed using 70% methanol for 10 min. It was then followed by staining with 0.2% crystal violet, washing with distilled water and air drying at room temperature [91]. Under an inverted microscope, invaded cells were then observed. The cells invasion through the permeable matrix gel was expressed as percentage of invading cells and calculated as:

% invading cells = [Mean number of cells invading through the permeable matrix gel/Mean number of cells migrating through uncoated culture insert] × 100(2)

Mean number of cells migrating through uncoated culture insert.

### 4.7. Cell Adhesion Assay 

The effect of *S. repanda* crude extract on the adhesion ability of A549 and HCT-116 cells was carried out according to the method described previously by Burg–Roderfeld et al., (2007) [92]. Firstly, 0.1% gelatin was coated on the surface of the 6 well culture plates and left to air dry at 37 °C for 30 min. Both A549 and HCT-116 cells (1 × 10^6^ cells/mL) were harvested by trypsin and resuspended in a medium with and without *S. repanda* crude extract (100–500 μg/mL). Then, cells were dispensed into the respective wells and further incubated at 37 °C for 2, 4 and 6 h in 5% CO_2_. At the end of incubation, attached cells were gently washed with PBS twice and counted time dependently under an inverted microscope. 

### 4.8. Expression Levels Determination of Metastasis Related Genes

Using the TriPure Isolation Reagent (Sigma-Aldrich^®^, India) and according to the manufacturer’s instructions, cellular RNA was isolated. RNA was quantified electrophoretically using 1.2% agarose gel, staining with ethidium bromide and visualizing under UV light. Firstly, RT-first strand synthesis kit (Qiagen^®^, CA, USA) was used to reverse transcribe the 1 μg of isolated RNA, and then the relative expression of metastasis related genes was determined by SYBR green based qRT PCR method (Applied Biosystems^®^ 7500 Fast Real-Time PCR machine, CA, USA). ΔΔCt method was then followed to analyze the data, and values were expressed in terms of fold change relative to control [93,94]. Cycling conditions for relative expression of genes were as follows: initial reverse transcription at 55 °C for 45 min, 1 cycle denaturation of 95 °C with 10 min hold, followed by 40 cycles of 95 °C with 15 s hold, annealing temperature at 60 °C (MMP-2, MMP-9, MT1-MMP, TIMP-1 and GAPDH) with a 60 s hold. Four pairs of primers were separately used (Table 2). Samples were run in triplicate and their relative expression was determined by normalizing the expression of each target GAPDH.

### 4.9. Statistical Analysis

All the results are expressed as mean ± SD of the number of experiments performed. A significance test was carried out among the treatments by one way ANOVA followed by Tukey’s post hoc test at *p* < 0.05. Statistical analysis was conducted with software GraphPad Prism 5.0.

## 5. Conclusions

As per our knowledge, this study is the first one that demonstrates the anti-metastatic effect of *S. repanda* crude extract on lung and colon cancer cells. In conclusion, this study revealed that *S. repanda* crude extract exerts an inhibitory effect on various crucial steps of metastasis such as cell adhesion, invasion and migration via modulating the activities of metastasis related proteases and their activators and inhibitors. This reveals that *S. repanda* can be used/recommended as a potential anti-metastatic agent for drug development and therapy in cancer treatment.

## Figures and Tables

**Table 1:** Phytochemical composition of *S. repanda* crude extract identified by HR-LC-MS technique [9].

Phytocompounds	Formula	Class	*m/z*	RT (min)	Mass
**Urocanic acid**	C_6_H_6_N_2_O_2_	monocarboxylic acid	131.2	24.845	138.04267
**Hordenine**	C_10_H_15_NO	alkaloid	167.2	23.071	169.23958
**Oleamide**	C_18_H_35_NO	fatty acid	286.1	20.177	281.27135
**Hexadecanamide**	C_16_H_33_NO	fatty acid amide	250.4	19.849	255.25575
**Arachidonic acid**	C_20_H_32_O_2_	polyunsaturated fatty acid	301.8	18.601	304.2397
**4-Coumaric acid**	C_9_H_8_O_3_	phenol	168.2	18.265	164.04718
**2-Arachidonoyl glycerol**	C_23_H_38_O_4_	fatty acid derivative	371.0	16.012	378.27635
**Valine**	C_5_H_11_NO_2_	amino acid	118.4	13.901	117.07901
**Genistein**	C_15_H_10_O_5_	isoflavone	275.3	13.542	270.05214
**Diosmetin**	C_16_H_12_O_6_	flavonoid	294.3	13.534	300.06279
**2-Amino-1,3,4-octadecanetriol**	C_18_H_39_NO_3_	phenol	311.5	13.499	317.29231
**Glycitein**	C_16_H_12_O_5_	flavonoid	288.9	13.477	284.06807
**Rhamnetin**	C16H12O7	flavonoid	311.2	12.452	316.05766
**Formononetin**	C_16_H_12_O_4_	phenol	265.3	12.157	268.07329
**Luteolin**	C_15_H_10_O_6_	flavonoid	278.6	11.735	286.04723
**Apigenin**	C_15_H_10_O_5_	flavone	272.4	11.177	270.05235
**Glycitin**	C_22_H_22_O_10_	isoflavone	442.2	11.088	446.12065
**(-)-Caryophyllene oxide**	C_15_H_24_O	epoxide	228.4	10.861	220.18227
**Kuromanin**	C_21_H_20_O_11_	pigment	458.1	8.976	448.09996
**Kaempferol**	C_15_H_10_O_6_	flavonoid	294.6	8.662	286.04726
**Sedanolide**	C_12_H_18_O_2_	isobenzofuran	199.5	8.578	194.13039
**α-Pinene-2-oxide**	C_10_H_16_O	terpenoid	148.9	8.494	152.11989
**Quercetin-3β-D-glucoside**	C_21_H_20_O_12_	flavonoid	260.3	8.438	464.09465
**Quercetin**	C_15_H_10_O_7_	flavonoid	308.6	8.414	302.04192
**Vitexin**	C_21_H_20_O_10_	flavonoid	436.8	8.319	432.10497
**Rutin**	C_27_H_30_O_16_	flavonoid	615.4	8.291	610.15239
**4-Methoxycinnamic acid**	C_10_H_10_O_3_	phenol	186.2	7.754	178.06275
**Norharman**	C_11_H_8_N_2_	alkaloid	164.7	6.725	168.06847
**4-Hydroxycoumarin**	C_9_H_6_O_3_	benzopyrone	158.8	6.568	162.0314
**Methyl cinnamate**	C_10_H_10_O_2_	cinnamic acid ester	164.5	6.458	162.06775
**Isoferulic acid**	C_10_H_10_O_4_	phenol	198.6	6.367	194.05762
**Scopoletin**	C_10_H_8_O_4_	coumarin	196.3	5.969	192.04198
**Citral**	C_10_H_16_O	terpenoid	145.8	5.575	152.11989
**Pulegone**	C_10_H_16_O	terpenoid	158.9	4.94	152.11989
**Caffeic acid**	C_9_H_8_O_4_	phenol	186.2	4.692	180.04181
**7-Hydroxycoumarine**	C_9_H_6_O_3_	phenol	169.4	4.651	162.0314
**Chlorogenic acid**	C_16_H_18_O_9_	phenol	360.5	4.646	354.09435
**Kynurenic acid**	C_10_H_7_NO_3_	quinoline carboxylic acid	181.7	3.823	189.04239
**Coumarin**	C9H6O2	phenol	148.6	3.784	146.0365
**3-Methylcrotonylglycine**	C_7_H_11_NO_3_	amino acid	152.6	3.325	157.0737
**4-Hydroxyphenylacetic acid**	C_8_H_8_O_3_	benzenoid	156.3	3.314	152.04714
**8-Hydroxyquinoline**	C_9_H_7_NO	alkaloid	153.2	2.965	145.05255
**Maltol**	C_6_H_6_O_3_	sugar	130.4	2.278	126.03161
**Guvacoline**	C_7_H_11_NO_2_	pyridine alkaloid	145.2	1.539	141.07878
**L-Phenylalanine**	C_9_H_11_NO_2_	amino acid	164.2	1.375	165.07883
**L-Norleucine**	C_6_H_13_NO_2_	amino acid	136.5	1.134	131.09453
**L-Pyroglutamic acid**	C_5_H_7_NO_3_	amino acid	133.1	1.04	129.0425
**D-Glucosamine**	C_6_ H_13_ NO_5_	amino sugar	183.3	0.946	179.079
**Betaine**	C_5_H_11_NO_2_	amino acid	122.2	0.935	117.07901
**L(-)-Carnitine**	C_7_H_15_NO_3_	amino acid derivative	157.6	0.93	161.10489
**Acetylcholine**	C_7_H_15_ NO_2_	essential nutrient (vitamin)	149.7	0.850	145.11
**α-Lactose**	C_12_H_22_O_11_	sugar	349.9	0.839	342.11521
**Choline**	C_5_H_13_N O	essential nutrient (vitamin)	111.4	0.798	103.09988

**Table 2:** List of primers used for metastasis related genes [95].

Sl. No	Primer	Sequence
1	**MMP-2**	**sense**–5′-GGCCCTGTCACTCCTGAGAT-3′ **antisense**–5′-GGCATCCAGGTTATCGGGGA-3′
2	**MMP-9**	**sense**–5′- CGGAGCACGGAGACGGGTAT-3′ **antisense**–5′- TGAAGGGGAAGACGCACAGC-3′
3	**MT1-MMP**	**sense**–5′-TGGGTAGCGATGAAGTCTTC-3′ **antisense**–5′-AGTAAAGCAGTCGCTTGGGT-3′
4	**TIMP-1**	**sense**–5′- GATCCAGCGCCCAGAGAGACACC-3′ **antisense**–5′-TTCCACTCCGGGCAGCATT-3′
5	**GAPDH**	**sense**–5′- CGAGATCCCTCCAAAATCAA-3′ **antisense**–5′-AGGTCCACCACTGACACGTT-3′

## Data Availability

All data generated or analyzed during this study are included in this article.
